# The Acute Effects of Leptin on the Contractility of Isolated Rat Atrial and Ventricular Cardiomyocytes

**DOI:** 10.3390/ijms23158356

**Published:** 2022-07-28

**Authors:** Anastasia Khokhlova, Tatiana Myachina, Xenia Butova, Anastasia Kochurova, Ekaterina Polyakova, Michael Galagudza, Olga Solovyova, Galina Kopylova, Daniil Shchepkin

**Affiliations:** 1 Institute of Immunology and Physiology, Russian Academy of Sciences, Pervomajskaya Str. 106, 620049 Yekaterinburg, Russia; myachina.93@mail.ru (T.M.); butchini@mail.ru (X.B.); kochurova.a.m@mail.ru (A.K.); o-solovey@mail.ru (O.S.); g_rodionova@mail.ru (G.K.); d.shchepkin@iip.uran.ru (D.S.); 2 Almazov National Medical Research Centre, Institute of Experimental Medicine, Akkuratova Str. 2, 197341 Saint-Petersburg, Russia; polyakova_ea@yahoo.com (E.P.); galagudza@almazovcentre.ru (M.G.)

**Keywords:** leptin, atria, ventricles, single cardiomyocytes, sarcomere shortening, calcium transients, actin–myosin interaction, protein phosphorylation

## Abstract

Leptin is a pleiotropic peptide playing an important role in the regulation of cardiac functions. It is not clear whether leptin directly modulates the mechanical function of atrial cardiomyocytes. We compared the acute effects of leptin on the characteristics of mechanically non-loaded sarcomere shortening and cytosolic Ca^2+^ concentration ([Ca^2+^]_i_) transients in single rat atrial and ventricular cardiomyocytes. We also studied the functional properties of myosin obtained from cardiomyocytes using an in vitro motility assay and assessed the sarcomeric protein phosphorylation. Single cardiomyocytes were exposed to 5, 20, and 60 nM leptin for 60 min. In ventricular cardiomyocytes, 60 nM leptin depressed sarcomere shortening amplitude and decreased the rates of shortening and relaxation. These effects were accompanied by a decrease in the phosphorylation of cMyBP-C, an increase in Tpm phosphorylation, and a slowdown of the sliding velocity of thin filaments over myosin in the in vitro motility assay. In contrast, in atrial cardiomyocytes, the phosphorylation of cMyBP-C and TnI increased, and the characteristics of sarcomere shortening did not change. Leptin had no effect on the characteristics of [Ca^2+^]_i_ transients in ventricular cardiomyocytes, while 5 nM leptin prolonged [Ca^2+^]_i_ transients in atrial cardiomyocytes. Thus, leptin-induced changes in contractility of ventricular cardiomyocytes may be attributed to the direct effects of leptin on cross-bridge kinetics and sarcomeric protein properties rather than changes in [Ca^2+^]_i_. We also suggest that the observed differences between atrial and ventricular cardiomyocytes may be associated with the peculiarities of the expression of leptin receptors, as well as signaling pathways in the atrial and ventricular myocardium.

## 1. Introduction

Leptin, a 16 kDa peptide hormone encoded by the ob gene, is one of the most important and widely studied factors in the control of energy balance [1,2]. Leptin plasma concentrations are elevated in obesity [3,4] as well as in cardiovascular diseases, independent of body weight, such as ischemic heart disease [5], cardiomyopathy [6], and congestive heart failure [7]. Leptin is produced mainly by the adipocytes but it also is secreted by cardiomyocytes [6,8,9]. The leptin receptors were found in cardiomyocytes [10,11], suggesting that leptin directly modulates the heart’s function and metabolism.

Leptin has complex effects on the heart’s function, including characteristics of action potential, Ca^2+^ handling, and the contractility of cardiomyocytes [1,12]. It was found that the acute treatment of rat ventricular cardiomyocytes with leptin inhibits their contraction [13,14,15]. On other hand, leptin deficiency in ob/ob mice caused a violation of the cardiomyocyte structure and impaired contractile functions and Ca^2+^ handling in ventricular myocytes [16]. The leptin receptor blockade improved left ventricular systolic function, and reduced the expression of genes associated with extracellular matrix remodeling in the postinfarcted rat heart [17] but the deletion of leptin receptors in the murine myocardium led to lethal heart failures [18]. An increase in leptin expression during myocardial infraction stimulated myocardial remodeling [5] and promoted local oxidative stress [19]. Leptin may have beneficial effects on the heart, providing protections against cardiac lipotoxicity [20] and hypertrophy caused by transverse aortic constriction [21].

However, the precise mechanism of leptin-induced cardiac contractile dysfunction in hyperleptinemic conditions is still unelucidated. Previous studies depicted that leptin depresses cardiac contractility either directly through mechanisms related to nitric oxide (NO) production [13], janus kinase/signal transducer and the activator of the transcription (JAK-STAT) and NADPH oxidase-dependent pathway [22], and p38 mitogen-activated protein kinase (MAPK) pathways [23] or indirectly via enhanced sympathetic nerve activity [15,24,25]. As for other aspects of cardiac contraction, attention to date has focused on ventricles; thus, little is known about these mechanisms in the atria.

Atria and ventricles differ in the expression of leptin and its receptors. Leptin mRNA expression was higher in the atria than in the ventricles of feline hearts [6]. The gene expressions of leptin and its receptors (OB-Ra, OB-Rb, and OB-Re) were also greater in the atria compared to ventricles in rat hearts [11]. The higher leptin concentrations in the atria than in ventricles suggest regional differences in myocardial reactivity to leptin. However, no comparative studies on the effects of leptin on functional properties of atrial and ventricular myocardium have been conducted.

The purpose of this study is to compare the direct effect of leptin on the contractile activity and cytosolic Ca^2+^ concentration ([Ca^2+^]_i_) transients in rat single atrial and ventricular cardiomyocytes. To explore potential mechanisms of leptin-induced differences in contractile response between atrial and ventricular cardiomyocytes, we studied the functional properties of cardiac myosin using an in vitro motility assay and assessed sarcomeric protein phosphorylation.

## 2. Results

### 2.1. The Effects of Leptin on Sarcomere Dynamics and [Ca^2+^]_i_ Transients in Single Atrial and Ventricular Cardiomyocytes

**Figure 1:**
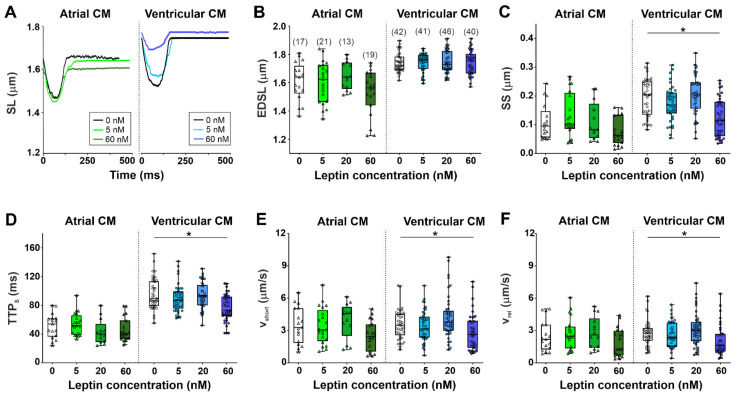
The acute effects of leptin on sarcomere shortening in single atrial and ventricular cardiomyocytes (CM). (**A**) Representative recordings of sarcomere length (SL) changes in mechanically non-loaded atrial and ventricular cardiomyocytes from the control group (0 nM) and after incubation with 5 and 60 nM leptin for ≈60 min. (**B**) End-diastolic sarcomere length (EDSL). (**C**) Sarcomere-shortening amplitude (SS). (**D**) Time to peak sarcomere shortening (TTP_S_). (**E**) Maximum sarcomere-shortening velocity (v_short_). (**F**) Maximum sarcomere relaxation velocity (v_rel_). Each dot represents an individual cell; the total number of cells from 6 hearts (for atrial cardiomyocytes) or from 8 hearts (for ventricular cardiomyocytes) is shown in parentheses above the first set of boxplots. Data are presented in box and whisker plots, where the boxes are drawn from Q1 to Q3, horizontal lines represent median values and whiskers provide the 100% range of the values. * *p* < 0.05 compared with the control group with the Kruskal–Wallis test.

Consistently with previous studies on rats [26,27,28], single atrial cardiomyocytes from the control group had shorter end-diastolic sarcomere length (EDSL), lower absolute and fractional sarcomere shortening amplitudes, shorter time to peak shortening (TTP_S_), and shorter time to 50% relaxation (TTR_50_) compared to ventricular cardiomyocytes (*p* < 0.0001, Mann–Whitney test, Figure 1, Figure S1 in Supplementary Materials). The maximum velocities of sarcomere shortening and relaxation (v_short_ and v_rel_) were not different between atrial and ventricular cardiomyocytes.

A representative trace depicting the effects of 5 and 60 nM leptin on sarcomere shortening in atrial and ventricular cardiomyocytes is shown in Figure 1A. Leptin did not affect EDSL and TTR_50_ (Figure 1B and Figure S1A). At a concentration of 60 nM, leptin reduced the sarcomere shortening amplitude by ≈44% of the control median value (*p* < 0.0001); decreased v_short_ and v_rel_ by ≈24 and 42%, respectively (*p* < 0.05); and decreased TTP_S_ by ≈20% (*p* < 0.0001) in ventricular cardiomyocytes; however, it did not affect these characteristics in atrial cardiomyocytes (Kruskal–Wallis test, Figure 1C–F and Figure S1B). The incubation of cardiomyocytes with 5 and 20 nM leptin did not exhibit any obvious effects on the characteristics of sarcomere dynamics either in ventricular or in atrial cardiomyocytes.

Thus, high leptin concentrations inhibited contraction amplitudes and decreased the rate of contraction and relaxation in ventricular cardiomyocytes, while it did not affect the contraction and relaxation of atrial cardiomyocytes.

**Figure 2:**
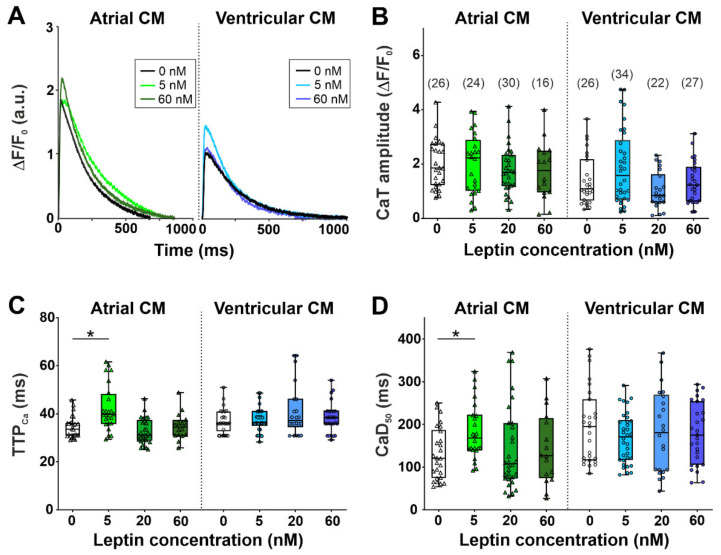
The acute effects of leptin on [Ca^2+^]_i_ transients in single atrial and ventricular cardiomyocytes (CM). (**A**) Representative recordings of [Ca^2+^]_i_ transients in mechanically non-loaded atrial and ventricular cardiomyocytes from the control group (0 nM) and after incubation with 5 and 60 nM leptin for ≈60 min. (**B**) [Ca^2+^]_i_ transient (CaT) amplitude. (**C**) Time to peak [Ca^2+^]_i_ transients (TTP_Ca_). (**D**) Time to 50% [Ca^2+^]_i_ transient decay (CaD_50_). Each dot represents an individual cell; the total number of cells from 6 hearts (for atrial cardiomyocytes) or from 8 hearts (for ventricular cardiomyocytes) is shown in parentheses above the first set of boxplots. Data are presented in box and whisker plots, where the boxes are drawn from Q1 to Q3, horizontal lines represent median values, and whiskers provide the 100% range of the values. * *p* < 0.05 compared with the control group with the Kruskal–Wallis test.

To study if the leptin-induced depression of sarcomere shortening in ventricular cardiomyocytes was associated with the reduced availability of [Ca^2+^]_i_, we examined the effects of leptin on the characteristics of [Ca^2+^]_i_ transients. In the control group, the amplitude of [Ca^2+^]_i_ transients was greater (*p* < 0.01), while time to peak [Ca^2+^]_i_ transients (TTP_Ca_) and time to 50% [Ca^2+^]_i_ transient decay (CaD_50_) were shorter (*p* < 0.05) in atrial myocytes compared to ventricular myocytes (Mann–Whitney test, Figure 2) as also previously shown in [28]. A shorter [Ca^2+^]_i_ transients in atrial cardiomyocytes compared to ventricular myocytes can be explained by shorter action potential, and both the lower expression of phospholamban [29,30] and enhanced SERCA2a activity [26,30]. The enhanced SERCA2a activity in the atrial myocardium may lead to higher sarcoplasmic reticulum Ca^2+^ loads in the atria than in ventricles [30,31] and, thus, explains the observed higher [Ca^2+^]_i_ transient amplitudes.

Leptin at a concentration of 5 nM prolonged TTP_Ca_ and CaD_50_ in atrial cardiomyocytes by 18% and 40%, respectively (*p* < 0.05), but did not affect the characteristics in ventricular cardiomyocytes (Kruskal–Wallis test, Figure 2). Other concentrations of leptin had no effect on the characteristics of [Ca^2+^]_i_ transients (Kruskal–Wallis test, Figure 2).

Thus, observed leptin-induced changes in the parameters of the contraction of ventricular cardiomyocytes were not easily associated with changes in [Ca^2+^]_i_. Atrial cardiomyocytes showed a non-linear response to increasing leptin concentrations with prolonged [Ca^2+^]_i_ transients at a near-physiological leptin concentration of 5 nM only.

### 2.2. The Effects of Leptin on Actin-Myosin Interaction and Sarcomeric Protein Phosphorylation in Atrial and Ventricular Cardiomyocytes

**Figure 3:**
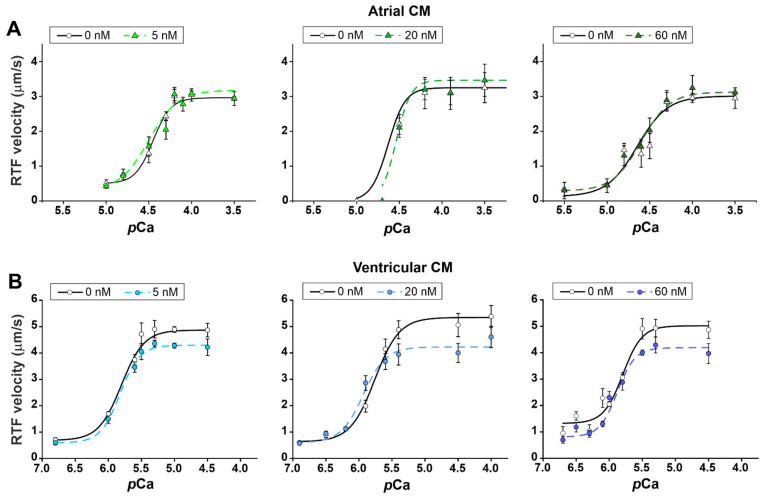
The acute effects of leptin on actin–myosin interactions in atrial (**A**) and ventricular (**B**) cardiomyocytes (CM). Ca^2+^-dependent sliding velocity of reconstructed thin filaments (RTF) over myosin in the in vitro motility assay from the control group (0 nM) and after incubation with leptin for ≈60 min. Each dot represents a mean value (30–100 filaments per heart, the heart number is 2 in each group). For each leptin concentration, the same hearts were used as the individual control. The experimental data are approximated by the Hill equation. The parameters of the Hill equation (v_0_, v_max_ and *p*Ca_50_) are shown in Table 1. Data are mean ± SD.

To reveal the effects of leptin on actin–myosin interaction, we studied the Ca^2+^-dependence of the sliding velocity of reconstructed thin filaments (RTF) over myosin from suspensions of atrial and ventricular cardiomyocytes using the in vitro motility assay. In the control group, the maximum sliding velocity of RTF (v_max_, the sliding velocity at saturating Ca^2+^ concentration) and the Ca^2+^ sensitivity of the velocity (*p*Ca_50_, *p*Ca, at which the sliding velocity is half-maximal) were higher over ventricular myosin than over the atrial one (*p* < 0.01, unpaired *t*-test, Figure 3).

We found that leptin decreased the v_max_ of RTF sliding over ventricular myosin by ≈16% (*p* < 0.05, unpaired *t*-test for comparison with individual control, Figure 3, Table 1) but it did not change it over atrial myosin. Leptin did not affect the Ca^2+^ sensitivity of *p*Ca-velocity dependence in each type of cells (*p* < 0.05, unpaired *t*-test, Table 1).

**Figure 4:**
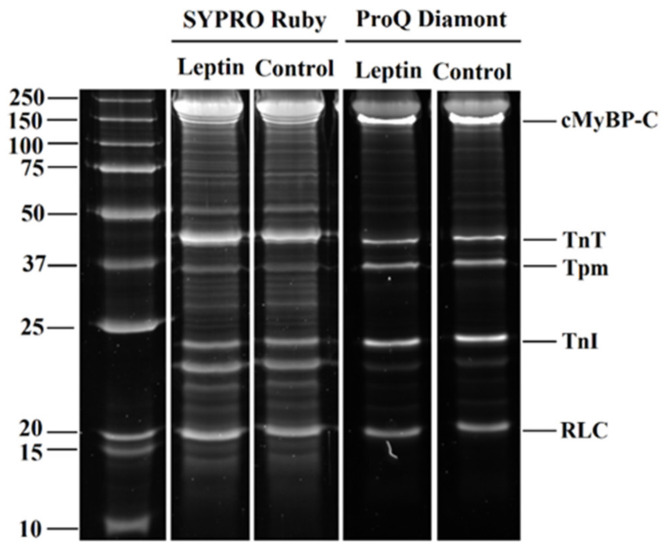
The example of gel electrophoresis of the sarcomeric protein extraction from ventricular cardiomyocytes of the control group (0 nM) and after incubation with 5 nM leptin for ≈60 min. cMyBP-C, cardiac myosin-binding protein-C; TnT, troponin T; Tpm, tropomyosin; TnI, troponin I; RLC, myosin regulatory light chain. Phosphorylation was assessed using Pro-Q Diamond and SYPRO Ruby (Invitrogen, Eugene, OR, USA). Precision Plus Protein™ Unstained Standards (Bio-Rad, Hercules, CA, USA) was used as molecular weight markers for proteins.

**Figure 5:**
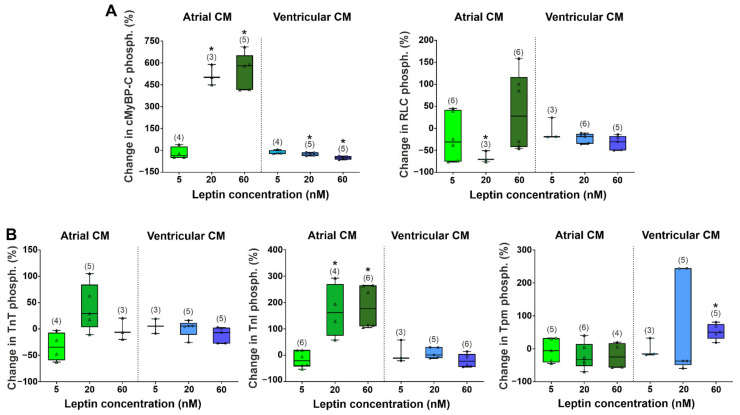
The acute effects of leptin on sarcomeric protein phosphorylation after incubation with leptin for ≈60 min. cMyBP-C, cardiac myosin-binding protein-C; RLC, myosin regulatory light chain; TnT, troponin T; TnI, troponin I; Tpm, tropomyosin. (**A**) Phosphorylation levels of cMyBP-C and RLC. (**B**) Phosphorylation levels of TnT, TnI, and Tpm. Phosphorylation is expressed as the ratio of the intensities of protein bands stained with Pro-Q Diamond and SYPRO Ruby (Invitrogen, Eugene, OR, USA) and then calculated as the % change related to the individual control values. The total number of protein samples for gel staining from 2 hearts is shown in parentheses. Data are presented in box and whisker plots, where the boxes are drawn from Q1 to Q3, horizontal lines represent median values and whiskers give the 100% range of the values.* *p* < 0.05 compared with the control group (0 nM leptin) with the Mann–Whitney test.

The phosphorylation of sarcomere proteins of cardiomyocytes is one of the important mechanisms for regulating myocardial contraction. We analyzed changes in the phosphorylation of regulatory light chain of myosin (RLC), cardiac myosin-binding protein C (cMyBP-C), troponin T (TnT), troponin I (TnI), and tropomyosin (Tpm) (Figure 4 and Figure 5). In the control group, the phosphorylation levels of cMyBP-C, RLC, TnI, and TnT were higher in ventricular cardiomyocytes compared to atrial cardiomyocytes (*p* < 0.05, Mann–Whitney U test, Figure 5A,B). Leptins at 20 and 60 nM decreased cMyBP-C phosphorylation in ventricular cardiomyocytes by 25 and 52%, respectively, but increased it in atrial cardiomyocytes (by 501%, *p* < 0.05 at 20 nM and by 580%, *p* < 0.01 at 60 nM, Mann–Whitney U test for comparison with individual control, Figure 5A). Leptins at 20 nM decreased RLC phosphorylation in atrial cardiomyocytes by 70% (*p* < 0.05). In ventricular cardiomyocytes, 60 nM leptin increased Tpm phosphorylation by 50% (*p* < 0.05). In atrial cardiomyocytes, 5 nM leptin did not affect TnI phosphorylation (*p* < 0.05), and 20 and 60 nM leptin increased it (by 161%, *p* < 0.05 and by 177%, *p* < 0.01, respectively, Figure 5B). Thus, the changes in sarcomeric protein phosphorylation were more prominent in atrial cardiomyocytes.

## 3. Discussion

In the current study, we compared, for the first time, the direct mechanical responses of the ventricular and atrial myocardium to increasing leptin concentrations and found that leptin has a complex effect on the contractility and [Ca^2+^]_i_ transients in rat single cardiomyocytes. The incubation of cardiomyocytes with leptin at a high concentration (60 nM) for 1 h depressed contractility of ventricular cardiomyocytes but did not affect the contractility of atrial cardiomyocytes. A low concentration of leptin (5 nM) prolonged [Ca^2+^]_i_ transients in atrial cardiomyocytes but did not affect these characteristics in ventricular cardiomyocytes. We also showed that leptin had a direct effect on the actin–myosin interaction. Leptin decreased the sliding velocity of thin filament over ventricular myosin in the in vitro motility assay and differently affected the phosphorylation of sarcomeric proteins in atrial and ventricular cardiomyocytes.

### 3.1. Effects of Leptin on the Contractility of Single Ventricular Cardiomyocytes

In ventricular cardiomyocytes, 60 nM leptin depressed sarcomere shortening and reduced v_short_ and v_rel_ (Figure 1), which is consistent with previous findings [13,14,15]. A decrease in vshort could be explained by the slow down of the cross-bridge kinetics of ventricular myosin, as evidenced by a decrease in the maximal sliding velocity of RTF over myosin in the in vitro motility assay (Figure 3; Table 1). In ventricular cardiomyocytes, leptin did not affect parameters of [Ca^2+^]_i_ transients. This result suggests that in ventricular cardiomyocytes, the leptin-induced attenuation of sarcomere shortening could not be explained by changes in [Ca^2+^]_i_.

Our results are in agreement with other studies showing that leptin concentrations less than 500 nM do not affect [Ca^2+^]_i_ transient amplitudes and decrease sarcomere shortening amplitude in ventricular cardiomyocytes [14,23]. In these studies, leptin concentrations higher than 500 nM depressed both sarcomere shortening and [Ca^2+^]_i_ transient amplitudes. At the same time, Nickola and co-authors found that leptin in the concentration range of 10–1000 nM reduced [Ca^2+^]_i_ transient amplitudes [13]. The inconsistencies with our results could be explained by different experimental conditions. We showed no leptin effects on the decay time of [Ca^2+^]_i_ transients that are in a good agreement with previous results [13,14,15,23]. Thus, the leptin-induced decrease in the rate of sarcomere shortening and the relaxation of ventricular cardiomyocytes could more likely be related to the direct effects of leptin on cross-bridge kinetics and sarcomeric protein properties rather than changes in [Ca^2+^]_i_.

Different mechanical responses in atrial and ventricular cardiomyocytes to leptin treatments were accompanied by differences in sarcomeric protein phosphorylation. It has been demonstrated that cMyBP-C and its phosphorylation play a major role in the pumping function of the heart [32,33]. We found that leptin reduced cMyBP-C phosphorylation in ventricular cardiomyocytes, which can contribute to the depression of sarcomere shortening [34,35]. Phosphorylated Tpm may regulate cardiac muscle relaxation dynamics, affecting the rate at which single actin–myosin bonds form and rupture [36]. Tpm phosphorylation slows cardiac muscle relaxation at the myofibril levels and in vivo hearts without altering its Ca^2+^ sensitivity [37,38]. Thus, an increase in Tpm phosphorylation by leptin treatments in ventricular cardiomyocytes may also contribute to a reduced rate of their relaxation.

It is known that hyperleptinemia in human and animals, including rats, leads to an increase in blood pressure, myocardial hypertrophy, impaired left ventricular function, and arrhythmias [12,39,40,41]. Previous studies showed that leptin directly depresses ventricular cardiomyocyte contraction through mechanisms related to NO-, JAK-STAT-, and MAPK-signaling pathways [13,22,23,42]. The diminished b3-adrenergic receptor (b3AR) signaling might be responsible for changes in NO production in leptin-treated ventricular cardiomyocytes via endothelial nitric oxide synthase (eNOS) and neuronal nitric oxide synthase (nNOS), resulting in a decrease in their contractility [43]. Leptin has been indicated to facilitate ROS generation in ventricular myocytes through ET-1 receptor and NADPH oxidase-mediated pathways [22]. On the other hand, ROS activates redox-regulated signaling enzymes (e.g., protein kinase C and protein kinase A), resulting in changes in protein phosphorylation [44,45]. In addition, leptin leads to the activation of phospholipase C gamma [46,47], which regulates the activation of protein kinase C that may contribute to altering cMyBP-C phosphorylation.

### 3.2. Effects of Leptin on the Contractility of Single Atrial Cardiomyocytes

In contrast to ventricular cardiomyocytes, leptin had no obvious effects on the characteristics of sarcomere shortening in atrial cardiomyocytes. It is known that an increased phosphorylation of cMyBP-C and TnI enhances myocardial contraction and relaxation [48,49,50,51]. In contrast to ventricular cardiomyocytes, in atrial cardiomyocytes, the leptin-induced phosphorylation of cMyBP-C and TnI increased while the characteristics of contraction did not change. These results can be explained by the peculiarities of the contractile apparatus of cardiomyocytes, as well as differences in [Ca^2+^]_i_ transients affecting the contractile behavior of atrial and ventricular myocardium [26,29,30,52]. Data on leptin effects on Ca^2+^ handling in atria are very limited. For the first time, we found a non-linear effect of leptin on the characteristics of [Ca^2+^]_i_ transients in atrial cardiomyocytes. Leptins at a concentration of 5 nM prolonged TTP_Ca_ and CaD_50_, while at a higher concentration, it did not affect [Ca^2+^]_i_ transients. We were able to find the only paper studying the effects of leptin on [Ca^2+^]_i_ transients in atrial cardiomyocytes. Using rabbit atrial cardiomyocytes, Lin and co-authors showed that 100 nM leptin decreased the amplitudes of [Ca^2+^]_i_ transients and Ca^2+^ content in the sarcoplasmic reticulum [53]. The authors also found that leptin-treated cardiomyocytes had increased action potential durations that could be attributed to a larger sodium current and a smaller ultra-rapid delayed rectifier potassium current [53]. Whether there are species-dependent effects of leptin on the parameters of action potential and [Ca^2+^]_i_ transients should be further investigated.

It has been shown in rats that leptin contributes to atrial fibrosis and angiotensin II-evoked atrial fibrillation [54]. In patients with paroxysmal atrial fibrillation, leptin levels were correlated with the parameters of cardiac autonomic function [55]. These findings, together with our results, suggest that chronic hyperleptinemia may disturb electrophysiological function, leading to changes in Ca^2+^ handling and then to an impaired contractile function of atrial cardiomyocytes. However, leptin signaling pathways in the atrial myocardium are unknown and needs to be studied.

## 4. Materials and Methods

### 4.1. Isolation of Rat Atrial and Ventricular Cardiomyocytes

The animals used in the present study were treated according to Directive 2010/63/EU of the European Parliament and the Guide for the Care and Use of Laboratory Animals published by the US National Institutes of Health (NIH Publication No. 85-23, revised 1985). The experimental protocol was approved by The Animal Care and Use Committee of the Institute of Immunology and Physiology. Unless otherwise noted, all chemicals and reagents were purchased from Sigma-Aldrich (St Louis, MO, USA).

Adult male Wistar rats at 10 weeks of age (250–300 g) were housed in the institutional vivarium with free access to food (Delta Feeds LbK 120 S-19, BioPro, Novosibirsk, Russia) and water. Single atrial and ventricular myocytes were isolated as previously described [56]. Briefly, rats were deeply anesthetized with an intramuscular injection of 2% Xylazine (1 mL/kg body weight, Alfasan, Woerden, Netherlands) and Zoletil-100 (0.3 mL/kg body weight, Virbac, Carros, France), heparinized (5000 IU/kg, Ellara, Pokrov, Russia), and euthanized by exsanguination. The heart was removed and perfused at 35 °C with a solution containing (in mM) the following: 140 NaCl, 5.4 KCl, 1.0 CaCl_2_, 1.2 MgSO_4_, 10 HEPES, 20.0 taurine, 5.0 adenosine, and 11.1 glucose (pH 7.35), supplemented by heparin sodium (10 IU/mL). Then, the heart was perfused with a nominally free Ca^2+^-high K^+^ solution containing (in mM): 115 NaCl, 14.0 KCl, 0.05 CaCl_2_, 0.3 EGTA, 1.2 MgSO_4_, 10 HEPES, 20.0 taurine, 5.0 adenosine, and 11.1 glucose (pH 7.25). After the heart stopped beating, the heart was perfused for 10 min (≈12 min in total). Then, the perfusate was changed to an enzyme solution containing collagenase type 2 (≈305 U/mg, Worthington Biochemical, Lakewood, NJ, USA) and protease XIV (≈3.5 U/mg) (in mM): 115 NaCl, 14.0 KCl, 0.025 CaCl_2_, 1.2 MgSO_4_, 10 HEPES, 20.0 taurine, 5.0 adenosine, 11.1 glucose, collagenase 0.80 mg/mL, and protease 0.06 mg/mL (pH 7.35) ≈20 min. Atria were injected by a high-concentrated enzyme solution (1.0 mg/mL collagenase and 0.06 mg/mL protease) for 10–15 min for a better digestion of the extracellular matrix. All solutions were equilibrated with 100% O_2_.

Then, the heart was transferred to a Petri dish to perform further perfusion by enzyme solutions using a syringe. Next, atria and ventricles were separated by cutting along the atrioventricular border and exposed to gentle mechanical disruption to disperse cardiomyocytes in a stopping buffer (in mM): 115 NaCl, 14.0 KCl, 0.025 CaCl_2_, 1.2 MgSO_4_, 10 HEPES, 20.0 taurine, 5.0 adenosine, 11.1 glucose, and bovine serum albumin 0.5 mg/mL (pH 7.35). Extracellular Ca^2+^ was added incrementally back to 1.8 mM. Isolated cardiomyocytes were maintained at room temperature in a modified Tyrode solution containing (in mM): 140 NaCl, 5.4 KCl, 1.8 CaCl_2_, 1.0 MgSO_4_, 10 HEPES, and 11.1 glucose (pH 7.35). Experiments were performed after allowing cardiomyocytes to rest for at least 30 min.

### 4.2. Experimental Protocol of Leptin Treatment

Low nanomolar levels of leptin below 10 ng/mL (<1 nM) in rat blood plasma are considered as the physiological range [57]. Previously, we showed that leptin plasma concentrations in intact male Wistar rats aged 11–12 weeks was 3.0–4.0 ng/mL [41]. A recent study demonstrated that plasma leptin levels do not reflect cardiac leptin synthesis in the myocardium and may not predict leptin-related cardiovascular morbidity [5]. Here we used leptin concentrations that are comparable to earlier studies [13,22,53]: “low”, 5 nM, “medium”, 20 nM, and “high”, 60 nM.

Recombinant rat leptin (R&D Systems, Minneapolis, MN, USA) was diluted in Tris-HCl buffer (pH 8) to 1 mg/mL according to the manufacturer’s instructions. Cardiomyocytes were incubated with leptin (5, 20, and 60 nM) in a modified Tyrode solution at 36 ± 1 °C. To study sarcomere dynamics and [Ca^2+^]_i_ transients, isolated cardiomyocytes were incubated for 50 min in flacon tubes using a shaking water bath (LSB12, Novosibirsk, Biosan, Russia). Directly before measurements, cells were transferred to an experimental chamber on the microscope stage with a modified Tyrode solution (36 ± 1 °C) containing leptin. The total time of cardiomyocyte incubation with leptin was 60 ± 10 min. Cardiomyocytes from the control group were incubated in a Tris-HCl buffer without leptin.

To study actin–myosin interactions and sarcomeric protein phosphorylation, atrial and ventricular cardiomyocyte suspensions from each heart were separated to leptin-treated (≈60 min of incubation) and control groups (“individual” control for each leptin concentration), and then cardiac myosin and protein samples for gel staining were prepared.

### 4.3. Measurements of Sarcomere Dynamics

Sarcomere dynamics in mechanically non-loaded atrial and ventricular cardiomyocytes were assessed using a confocal laser scanning microscopy system (LSM 710, Carl Zeiss, Jena, Germany), as described previously [58]. In brief, myocytes were placed in a chamber on the microscope stage and superfused with a modified Tyrode solution. The cells were field stimulated with suprathreshold voltage at a frequency of 1 Hz using a pair of graphite electrodes on the opposite sides of the chamber connected to a Myopacer stimulator (IonOptix Corporation, Milton, MA, USA). For measurements of sarcomere length (SL), the image intensity profile in a selected narrow area (3 pixel high), horizontally oriented along a cell long axis, was recorded every 1–3 msec in the optical channel. To capture changes in SL during cell contraction/relaxation, a custom-made software (EqapAll6) was used [59,60]. Cells with obvious sarcolemmal blebs or spontaneous contractions were not used for mechanical recording.

Sarcomere shortening and relaxation were assessed using the following parameters: end-diastolic SL (EDSL), absolute sarcomere shortening amplitude (SS = EDSL minus end-systolic SL), fractional sarcomere shortening amplitude (SS/EDSL×100%), time from the onset of sarcomere shortening to peak shortening (time to peak shortening, TTP_S_), time from peak shortening to 50% sarcomere relaxation (TTR_50_), and maximum sarcomere shortening and relaxation velocities (v_short_ and v_rel_).

### 4.4. Measurements of [Ca^2+^]_i_ Transients

A separate group of myocytes was loaded with 1.7 µM Fluo-8 AM (AAT Bioquest, Sunnyvale, CA, USA) and 0.1% Pluronic^®^ F-127 (AAT Bioquest, Sunnyvale, CA, USA) for 20 min at room temperature in darkness and then washed with a modified Tyrode solution. The intensity of emitted fluorescence excited optically at 488 nm was collected at 493–575 nm using LSM 710. The change in the fluorescence intensity (ΔF/F_0_, where F_0_ is the initial fluorescence) was calculated and used as an index of changes in [Ca^2+^]_i_. The amplitude of [Ca^2+^]_i_ transients (CaT amplitude), time from start of [Ca^2+^]_i_ increase to peak systolic [Ca^2+^]_i_ (time to peak [Ca^2+^]_i_ transients, TTP_Ca_), and the time from TTP_Ca_ to 50% decay of [Ca^2+^]_i_ transients (CaD_50_) were assessed using EqapAll6.

### 4.5. Protein Preparation and Phosphorylation Analysis

Sarcomeric proteins were obtained using the suspension of single cardiomyocytes. For in vitro motility assay experiments, cardiac myosin was extracted from cardiomyocyte suspensions according to Margossian and Lowey [61] with modifications. F-actin and troponin were obtained from the bovine left ventricle [62,63]. Human a-tropomyosin (Tpm) was expressed in BL21(DE3) bacterial cells [64]. Regulated thin filaments (RTF) were reconstituted from F-actin, troponin, and Tpm in a flow chamber.

Protein phosphorylation was analyzed using a 12% SDS-PAGE with Pro-Q Diamond phosphoprotein staining (Invitrogen, Eugene, OR, USA). SYPRO Ruby (Invitrogen, Eugene, OR, USA) staining was used to estimate the amount of total proteins. Protein samples and gel staining were prepared according to manufacturer’s manual. The gel was imaged on the ChemiDoc MP Imaging System (Bio-Rad, Hercules, CA, USA), and band densities were determined with Image Lab 5.2.1 software (Bio-Rad, Hercules, CA, USA). A level of protein phosphorylation was expressed as a ratio of the Pro-Q Diamond intensity to the SYPRO Ruby intensity.

### 4.6. In Vitro Motility Assay

The in vitro motility assay experiments were described in detail previously [64]. Briefly, 500 µg/mL myosin in an AB buffer (in mM) (25 KCl, 25 imidazole, 4 MgCl_2_, 1 EGTA, and 20 DTT (pH 7.5)) containing 0.5 M KCl was loaded into the flow chamber with nitrocellulose inner surface. After 2 min, 0.5 mg/mL BSA was added for 1 min. A further 50 µg/mL of non-labelled F-actin in the AB buffer with 2 mM ATP was added for 5 min. TRITC-phalloidin labelled RTFs at a concentration of 10 nM (by G-actin) were added for 5 min. Unbound filaments were washed out with AB buffer. Finally, the chamber was washed with AB buffer containing 0.5 mg/mL, BSA, oxygen scavenger system, 20 mM DTT, 2 mM ATP, 0.5% methylcellulose, 100 nM Tn/Tpm, and appropriate Ca^2+^/EGTA in proportions was calculated with the MAXCHELATOR program (http://www.stanford.edu/~cpatton/webmaxc/webmaxcS.htm, accessed on 8 July 2021). The experiments were performed at 30 °C.

The sliding velocities of 30–100 filaments were measured using the GMimPro software [65]. All experiments were repeated three times and the individual experimental means were fitted to the Hill equation: v = v_max_(1 + 10*^h^*^∙(*p*Ca−*p*Ca50)^)^−1^, where v and v_max_ are the sliding velocity and the maximum velocity at saturating [Ca^2+^], respectively, *p*Ca_50_ (i.e., Ca^2+^ sensitivity) is *p*Ca (−lg[Ca^2+^]) at which the half-maximal velocity is achieved, and *h* is the Hill coefficient.

### 4.7. Statistical Analysis

Data analyses were carried out (Microsoft Corp, Redmond, WA, USA), Origin 8.0 (Origin Lab, Northampton, MA, USA), and GraphPrism 8.0 (GraphPad Software, San Diego, CA, USA). Data are expressed as the mean ± SD or median (interquartile range). Statistical comparisons of two groups were made by Student’s two-tailed *t*-test with Welch’s correction (parametric analysis) or Mann–Whitney U test (non-parametric analysis). When comparing multiple groups, we performed Kruskal–Wallis test (non-parametric analysis) with Dunn’s post hoc test. For parametric statistical analyses, data were distributed normally (checked using Shapiro–Wilk normality tests). The characteristics of *p*Ca-velocity dependence and sarcomeric protein phosphorylation for each leptin concentration were compared with the individual control values. A *p*-value of <0.05 was considered to indicate a significant difference between the parameters.

## 5. Summary

We found that single atrial and ventricular cardiomyocytes had different mechanical responses to acute leptin treatment that was accompanied by leptin-induced differences in the functional properties of cardiac myosin and sarcomeric protein phosphorylation. The differences between atrial and ventricular cardiomyocytes may be associated with the peculiarities of the expression of leptin receptors, as well as signaling pathways in the atrial and ventricular myocardium. Leptin signaling pathways in the atrial myocardium are poorly understood, and their role in the regulation of the function of sarcomeric and Ca^2+^ handling proteins remains unknown and needs to be studied.

The findings of this study point to the necessity of different preventive, diagnostic, and therapeutic approaches against hyperleptinemia-associated changes in atrial and the ventricular myocardium.

## 6. Limitations

We acknowledge several methodological limitations in this study. We studied direct leptin effects in vitro, while the systemic administration of leptin may lead to different results. Regarding contractility effects, we found that leptin was effective only at a high concentration of 60 nM, which is related to severe obesity. However, obesity is also associated with leptin resistance. On the other hand, in the present study, leptin was applied for a relatively short period of time (60 min) in cardiomyocytes isolated from control rats with presumably intact leptin signaling. A longer incubation time and pathological models might yield distinct results. These issues should be considered while interpreting the results. In addition, the underlying molecular mechanisms for the Ca^2+^ handling effects were beyond the scope of this paper, and future studies in this area are desirable.

## Figures and Tables

**Table 1:** The effects of leptin on the parameters of *p*Ca-velocity dependence obtained in the in vitro motility assay.

Origin of Myosin	Leptin Concentration (nM)	v_max_ (µm/s)	v_0_ (µm/s)	*p*Ca_50_
Atrial CM	0	3.0 ± 0.1	0.5 ± 0.1	4.45 ± 0.04
	5	3.2 ± 0.2	0.3 ± 0.2	4.50 ± 0.06
	0	3.3 ± 0.1	0	4.63 ± 0.03
	20	3.5 ± 0.1	0	4.54 ± 0.05
	0	3.0 ± 0.3	0.1 ± 0.1	4.64± 0.10
	60	3.1 ± 0.1	0.3 ± 0.2	4.60 ± 0.04
Ventricular CM	0	4.9 ± 0.1	0.7 ± 0.1	5.80 ± 0.03
	5	4.3 ± 0.1 *	0.6 ± 0.1	5.82 ± 0.06
	0	5.3 ± 0.2	0.6 ± 0.1	5.76 ± 0.06
	20	4.2 ± 0.2 *	0.6 ± 0.1	5.92 ± 0.08
	0	5.0 ± 0.2	1.3 ± 0.2	5.80 ± 0.06
	60	4.2 ± 0.2 *	0.8 ± 0.1	5.88 ± 0.04

v_max_ and v_0_, the maximum sliding velocity of thin filaments at saturating Ca^2+^ concentration and sliding velocity of filaments at low Ca^2+^ concentration, respectively; *p*Ca_50_, *p*Ca, at which the sliding velocity is half-maximal (Ca^2+^ sensitivity of sliding velocity); 30–100 filaments per heart, and the heart number is 2 in each group. Data are mean ± SD. * *p* < 0.05 compared with the control group (0 nM leptin), unpaired *t*-test.

## Supplementary Materials

The supporting information can be downloaded at: https://www.mdpi.com/article/10.3390/ijms23158356/s1.
