# Supplementary material for: The Acute Effects of Leptin on the Contractility of Isolated Rat Atrial and Ventricular Cardiomyocytes
Source: Int J Mol Sci. 2022 Jul 28;23(15):8356. doi: 10.3390/ijms23158356 (PMC9369024; doi:10.3390/ijms23158356)
Supplement: Supplementary file 1 [file ijms-23-08356-s001.zip › ijms-1828626-supplementary.pdf]

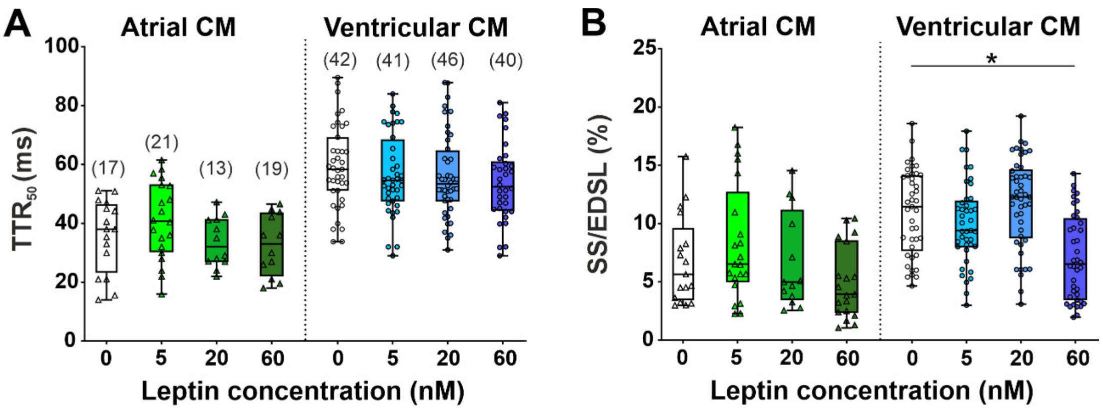

Figure S1. The acute effects of leptin on sarcomere shortening in single atrial and ventricular cardiomyocytes (CM). **(A)** Time to 50% relaxation (TTR_{50}). **(B)** Fractional sarcomere shortening amplitude (SS/EDSL×100%). Each dot represents an individual cell; the total number of cells from 6 hearts (for atrial cardiomyocytes) or from 8 hearts (for ventricular cardiomyocytes) is shown in parentheses above the first set of boxplots. Data are presented in box and whisker plots, where the boxes are drawn from Q1 to Q3, horizontal lines represent median values and whiskers give the 100% range of the values. *$p < 0.05$ compared with the control group (0 nM leptin), Kruskal–Wallis test.
